# A double-blind, randomised cross-over study to evaluate the absorption of a commercially available *Ginkgo biloba* extract compared to the liposomal extract Ginkgosome

**DOI:** 10.1186/s12906-022-03679-x

**Published:** 2022-08-03

**Authors:** David Briskey, Amanda Rao

**Affiliations:** 1 RDC Clinical, Newstead, Brisbane, QLD Australia; 2 School of Human Movement and Nutrition Sciences, University of Queensland, Brisbane, QLD Australia

**Keywords:** *Ginkgo biloba*, Absorption, Ginkgosome, Pharmacokinetics

## Abstract

**Background:**

*Ginkgo biloba* extracts (GBE) have been used in traditional medicines for centuries. GBE has been shown to deliver protective effects against symptoms of age-related cognitive decline. Despite there being standardised extractions for GBE, there is still variability in the absorption and efficacy of different extracts. Following the development of a liposomal GBE (Ginkgosome™), the aim of this study is to investigate the absorption of the liposomal formulation compared to a comparator formulation of equal dose.

**Methods:**

Thirteen healthy male and female volunteers completed this single equivalent dose, randomised, double-blind crossover study. Plasma concentrations were determined at baseline and at regular intervals over a 24-h period following ingestion of 120 mg of either a liposomal or comparator formulation.

**Results:**

The liposomal formulation was able to increase plasma concentration of ginkgolide B and C by 1.9 and 2.2-fold compared to the comparator formulation.

**Conclusion:**

The novel liposomal formulation is safe in humans and demonstrates superior absorption for the supply of GBE constituents compared to a comparator standardised formulation.

## Background

*Ginkgo biloba* (Ginkgoaceae) is a species of tree reported to have existed for millions of years [1, 2]. Extracts from the *Ginkgo biloba* tree have been used in traditional medicines for hundreds of years [3]. Today, *Ginkgo biloba* is considered endangered in parts of the world. In recent years the scientific evidence for the use of *Ginkgo biloba* extracts (GBE) has begun to establish its efficacy in several health areas. A main area of interest for GBE research has focused on its beneficial effects for reducing the symptoms of age-related cognitive decline, including mild memory impairment, cerebral insufficiency and dementia including Alzheimer’s disease [4–8]. GBE may also protect from the damaging effects of ischaemic events including neuronal degeneration [9, 10]. As such, sustainable practices are vital for the preservation/conservation of the *Ginkgo biloba* tree while allowing for the continued production of products using *Ginkgo biloba*.

The major constituents of GBE are the flavonoids and terpene trilactones (TTL) [11, 12] with the amount of each constituent within the plant possibly varying depending on conditions [13]. Therefore, standardised extracts have been established for use in evidence-based medicine and clinical trials [14]. Standardised GBE forms have specific concentration limits for the TTL fraction [ginkgolide A (GA), B (GB), C (GC), J (GJ) and bilobalide (BB)] of approximately 6% (GA, GB and GC combined totalling 2.8–3.4% and BB alone 2.6–3.2%), and the flavonoids fraction of approximately 24% of the extract [14, 15].

Despite the establishment of standardised GBE supplements, there is vast variability in the absorption and dissolution for extracts from different sources [15, 16]. Optimising absorption is an important step to help ensure the efficacy of GBE. To aid absorption, delivery methods are being incorporated which may include self-emulsifying drug delivery systems, cold water dispersion agents and liposomal formulations. Liposomal formulations as used in this study, involve encasing the supplement inside fat particles that are readily absorbed across transmucosal membranes, bypassing the digestive system to deliver the encapsulated compound intracellularly [17]. In the present study, a commercially available formulation of equal dose has been compared to a liposomal formulation. The primary aim of this study is to assess the total absorption profile [area under the curve (AUC)], concentration max (C_max_) and time to maximum concentration (T_max_) of 2 different GBE formulations in healthy participants over 24-h. Secondary aims assess product tolerability and therapeutic claims. It is hypothesised that the liposomal formulation will have a greater total absorption over 24 h compared to the standard, non-liposomal formulation.

## Material and methods

### Study design and procedures

A single equivalent dose, randomised, double-blinded, cross-over study conducted in Brisbane, Australia, was used to evaluate the bioavailability of 2 different GBE formulations administered as a single 120 mg dose. During the first clinic visit, participants were randomly allocated to 1 of 2 groups. Group 1: 480 mg liposomal Ginkgo (Ginkgosome™) containing 120 mg *Ginkgo biloba* extract (equivalent to 6 g dry leaf standardised to contain 24 mg of flavonglycosides 3.12 mg bilobalide 3.36 mg ginkgolides) or Group 2 (comparator): 120 mg of commercially available *Ginkgo biloba* extract (equivalent to 6 g dry leaf standardised to contain 29.4 mg flavonglycosides, 3.5 mg bilobalide and 3.7 mg ginkgolides). The Ginkgosome™ and comparator products were manufactured by different manufacturers and had a minimum of 12 months before product expiry date.

Upon completion of the first GBE testing, participants underwent a 2-week washout before returning to the clinic to repeat the trial under identical conditions for the second GBE formulation. The liposomal formulation was supplied by Network Nutrition (part of IMCD) and the comparator was purchased from a retail pharmacy off the shelf in Brisbane, Australia. The specific comparator product was selected as it contained the same amount of GBE per unit (120 mg) as the investigational liposomal product and is a product extensively published in the current literature. All study products used in this study were cultivated from sustainable sources dedicated to growing commercially available *Ginkgo biloba* leaf. As such, this study had no negative effect on the species population or distribution of *Ginkgo biloba*. This study was registered on the Australian New Zealand Clinical Trials Registry (ANZCTR) on 08/07/2019 (registration number ACTRN12619000953134) and conducted in accordance with ethics approval from The University of Queensland human ethics board (approval number 2018002290).

### Subjects

All participants involved in the study provided written informed consent. All participants were healthy males and females, aged 18–40 years, within healthy BMI range (18.5–25) and no history or evidence of clinically significant medical conditions. Participants were excluded if they reported any clinically significant medical condition; use of GBE and/or antioxidants within the past 3 months; current use of prescription medications except the oral contraceptive pill if female; and known allergy to GBE or other antioxidants.

All participants were provided with a list of foods to avoid for 48-h prior to the study date and were required to fast (water allowed) for 10-h prior to the collection of the first blood sample. Following the collection of a baseline blood sample, participants were dosed with their allocated trial product. Within 30 min of dosing, a standardised breakfast was supplied to participants. During each clinic visit (first 12 h of sample collection), participants were provided with standardised, nutritionally balanced meals (breakfast, lunch, and dinner) and snacks. The same foods were supplied to participants for both portions of the trial. Participants were required to remain at the clinic for the duration of the first 12-h of sample collection. During each clinic visit, safety of the product was assessed by monitoring participants and reporting any side effects experienced.

### Sample collection

GBE absorption was analysed from venous blood samples taken prior to supplementation (t = 0), followed by intervals of 1, 2, 3, 4, 5, 6, 8, 10, 12 and 24 h post supplementation. Samples were obtained from a vein in the antecubital fossa using a Cannula (BD, New Jersey) and 6 mL vacutainer containing lithium heparin (BD, New Jersey). Collected samples were immediately centrifuged at 4 °C for 10 min (2,000 × g). Once spun, separated plasma was aspirated and temporarily stored at -20 °C before being stored at -80 °C to await analysis.

### Sample analysis

GBE absorption was assessed by analysis of plasma for the metabolites: BB, GA, GB and GC. Analysis was conducted using liquid-chromatography tandem mass spectrometry (LC–MS/MS; Thermo Fisher Scientific) using a method based on previously published studies [18, 19]. Briefly, 400 µL of plasma, 600 µL of ethyl acetate and 100 µL of 3 M hydrochloric acid was added to a 2 mL microfuge tube and vortex mixed. The resulting solution was centrifuged for 10 min at 16,000 × g before the supernatant was transferred to a glass culture tube and dried under a stream of nitrogen gas at 30 °C. Once dry, the residue was reconstituted in 150 µL of methanol/water (1:1 v/v) and transferred to a vial where 20 µL was injected into the LC–MS/MS. Freshly prepared standards were analysed along with samples each day. A random selection of supplements from each group were analysed for concentrations along with the samples. The concentration of BB, GA, GB, and GC were calculated using analytical standards purchased from Sapphire Bioscience (Redfern, NSW, Australia). The assay was validated by running 10 replicates of a pool of plasma with and without spiking with known levels of BB, GA, GB, and GC.

Chromatography was performed using a Kinetex 5 µm C18, 250 × 4.6 mm with an AQ C18 4 × 3 mm SecurityGuard cartridge (Phenomenex, Torrance, CA) maintained at 40 °C. Mobile phase A consisted of water with 0.2% formic acid and mobile phase B consisted of acetonitrile with 0.2% formic acid. Mobile phase was run with an isocratic flow of 70% mobile phase B at a rate of 0.2 mL/min. A turbo ion spray interface was used operated in negative ion mode. Acquisition was made in multiple reaction monitoring mode using the following ions: BB 325 → 163, GA 407 → 319, GB 423 → 367 and GC 439 → 383.

### Statistical analysis

Any endogenous BB, GA, GB, or GC detected in the plasma at baseline will be subtracted from the plasma concentrations for all post-supplementation timepoints. Any values presented for AUC or C_max_ will have any endogenous concentrations already factored in the data to ensure any plasma concentration of BB, GA, GB, or GC detected is due to the supplement.

Statistical analysis was conducted using AUC values (0–24 h) calculated by the trapezoidal model. C_max_ and T_max_ were analysed from the average of each participant’s specific C_max_ and T_max_ from the participant’s individual absorption curve for each compound (as presented in Table 2). T-tests were performed to identify statistical difference between groups. All analysis was conducted using GraphPad Prism version 8 for Windows, GraphPad Software, La Jolla California USA, www.graphpad.com.

This study was powered to assess superiority of absorption based on a 75% response rate in the standard group compared to the active group, with similar standard deviations, with a 95% power. Based on these parameters, the number of subjects required to complete each arm was twelve. Allowing for dropouts, this study recruited fourteen people.

## Results

There was no statistical difference in participant demographics at baseline (Table 1). Of the fourteen participants recruited to take part in this study, thirteen completed both arms of the study. One participant was unable to complete the crossover portion of the study due to COVID-19 restrictions preventing their attendance. No adverse events or reactions were reported by any participant for either product.

**Table 1:** Participant demographics at baseline

Males (%)	6 (43)
Females (%)	8 (57)
Age (years)	31.2 ± 4.9

Data presented are mean ± SD

This study focuses on the metabolites BB, GB and GC, with all samples returning GA concentrations below the limit of detection. No endogenous BB, GB, GC or GA were found in any of the baseline samples.

**Fig. 1:**
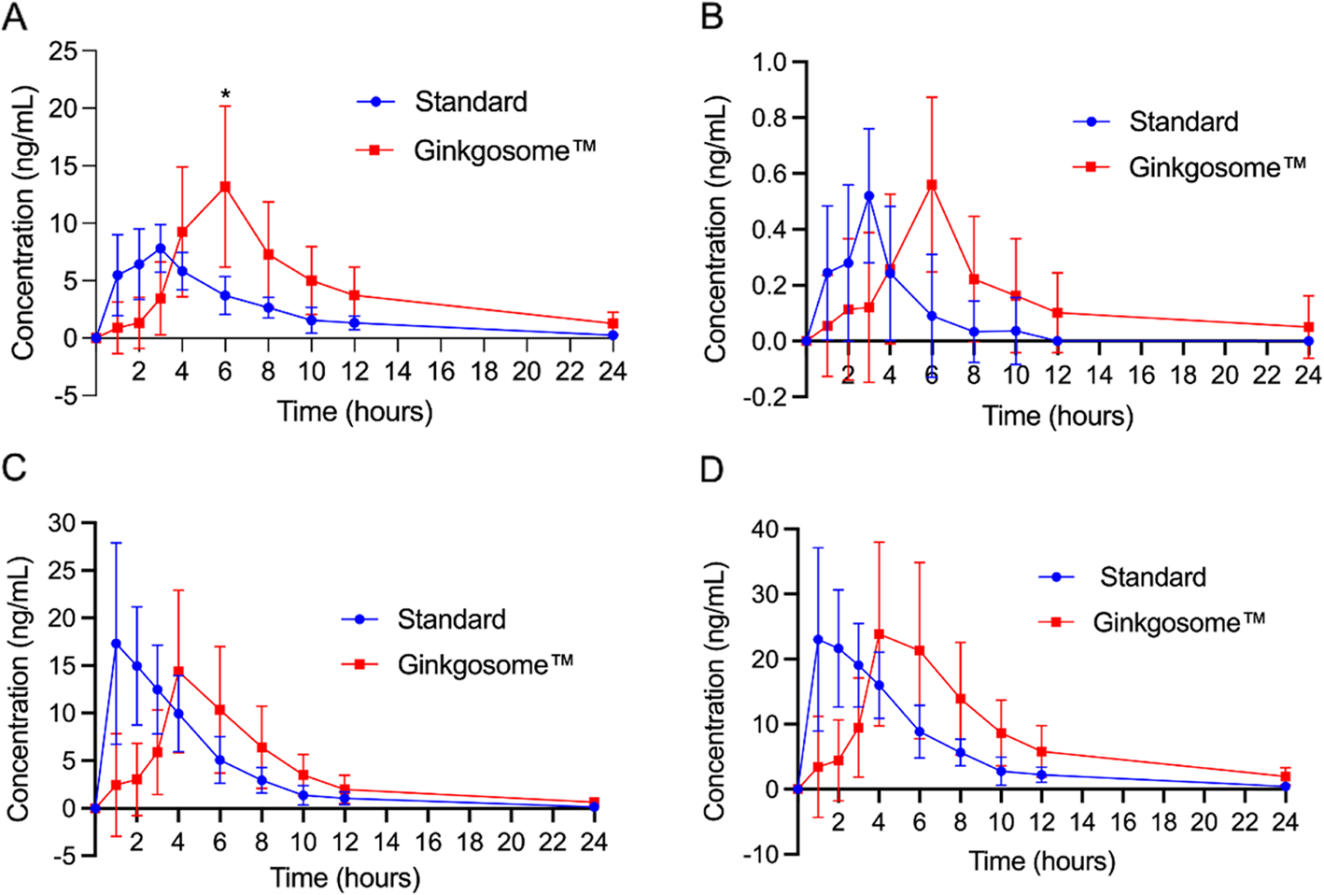
**A** Plasma absorption of GB. **B** Plasma absorption of GC. **C** Plasma absorption of BB. D) Total (GB, GC & BB) plasma absorption over 24 h. * Significantly different C_max_ p < 0.05. Data presented is the groups average for each specific time point and may not represent the true C_max_ and/or T_max_ as presented in Table 2

For GB and GC, the 120 mg Ginkgosome™ group had a 1.9 and 2.2-fold greater absorption (AUC) and a 1.7 and 1.2-fold greater C_max_ than that of the standard 120 mg formulation (*p* < 0.05; Table 2, Fig. 1A and B). For BB and total TTL, the Ginkgosome group showed equivalent absorption to the standard formulation (Table 2, Fig. 1C and D).

**Table 2:** GBE plasma absorption concentration, maximum plasma concentration and time to maximum concentration for GB, GC and BB for both groups. Data presented is the groups average based on the individual participants specific AUC, C_max_ and T_max_ data

	GB		GC		BB	
	Ginkgosome™	Standard	Ginkgosome™	Standard	Ginkgosome™	Standard
AUC	99.6 ± 52.8*	52.7 ± 17.7	4.2 ± 2.5*	1.9 ± 1.4	91.4 ± 38.8	86.6 ± 33.0
C_max_ (ng/mL)	13.2 ± 6.2*	7.8 ± 2.1	0.6 ± 0.1	0.5 ± 0.1	17.2 ± 7.1	19.6 ± 7.9
T_max_ (hrs)	4.6 ± 1.3*	2.5 ± 1.1	5.1 ± 1.2*	2.6 ± 1.6	4.5 ± 1.8*	1.8 ± 1.1

Values presented are values are mean ± SD

*GB* ginkgolide B, *GC* ginkgolide C, *BB* bilobalide, *AUC* area under the curve, *C*_*max*_ maximum concentration, *T*_*max*_ time for maximum concentration

^*^p < 0.05

Analysis of the trial products showed each supplement contained little to no GA (Table 3).

**Table 3:** Supplement analysis for GBE constituents for both groups

	Standard (µg/mg GBE)	Ginkgosome™ (µg/mg GBE)
BB	0.36 ± 0.03	0.26 ± 0.03
GA	BLD	BLD
GB	0.19 ± 0.02	0.28 ± 0.03
GC	0.17 ± 0.03	0.14 ± 0.02

Values presented are values are mean ± SD

*BLD* below limit of detection, *BB* bilobalide, *GA* ginkgolide A, *GB* ginkgolide B, *GC* ginkgolide C

When AUC data was corrected to be relative to the Ginkgosome™ group dose, GB and GC remained significantly different, and BB became significantly different (Table 4).

**Table 4:** GBE plasma absorption concentration for GB, GC and BB for both groups when corrected for capsule content. Data presented is the groups average based on the individual participants specific AUC data

	GB		GC		BB	
	Ginkgosome™™	Standard	Ginkgosome™	Standard	Ginkgosome™	Standard
AUC	99.6 ± 52.8*	71.9 ± 17.2^a^	4.2 ± 2.5*	1.6 ± 1.1	91.4 ± 38.8*	62.3 ± 23.7

Values presented are values are mean ± SD

*GB* ginkgolide B, *GC* ginkgolide C, *BB* bilobalide, *AUC* area under the curve

^a^one outlier (> 2SD from mean) was removed

^*^p < 0.05

## Discussion

Overall, the results of this study show Ginkgosome™ is safe and able to increase the absorption of GB and BB. The results are in keeping with other studies that have investigated methods to increase the absorption of GBE. Mauri and colleagues [18] supplemented healthy adults with 160 mg of either a standard formulation or a phospholipid complex formulation. Following the collection of blood samples over 400 min, Mauri and colleagues showed the complex formulation increased GB and BB AUC by approximately 2.45 and 2.0-fold respectively. The results of the present study and that of Mauri [18] are in contradiction to an early pharmacokinetic study conducted by Moreau and colleagues (1986) [20] who reported an absorption of a radiolabelled GBE of at least 60%. However, this study was conducted in rats, and the absorption efficiency in humans may be different.

One of the greatest differences observed in this study compared to other studies on GBE is the apparent lack of GA. Regardless of the difference in the individual ginkgolide reported in this study, when the results were normalised for each individual ginkgolide concentration (Table 4), the significant differences for AUC were maintained, and even the AUC for BB became significant. This further highlights the superior absorption of Ginkgosome™.

To help confirm our finding of no GA, a random selection of supplements from each group were analysed on multiple occasions to determine the concentration provided to participants. The analysis of the supplements confirmed the supplements appeared to contain no GA which is the likely reason for there being no GA seen in the blood. The atomic mass used in this study for GA was similar to that published by Tang and Colleagues (2008) [19] and Yuan and colleagues (2013) [21], but different to that published by Woelkart and colleagues (2010) [22] and Li and colleagues (2015) [23]. With GA having an atomic mass of 408.4 and the standard material used in this study being a certified analytical standard, there is no reason to suspect its accuracy of the mass used within this paper. Furthermore, the atomic mass used here for GB, GC, and BB is the same as those used by Yuan [21], Woelkart [22] and Li [23].

Another factor to consider when comparing studies is the product itself, as demonstrated by the formulations used in this study appearing to contain little or no GA (Table 3). This is supported by Kressmann and colleagues (2002) [16] who conducted a study comparing two different products with a 120 mg dose. Kressmann showed that there was a significant difference in absorption (AUC) of approximately 1.44, 3.15 and 1.13 for GA, GB, and BB respectively for two similar GBE formulations. This effect could be due to one of two reasons. Firstly, each product could contain vastly different amounts of each GBE constituent and secondly, each product and even each GBE constituent could absorb differently. When a trial product is quantified for TTL concentration, the quantification may not be conducted using mass spectrometry but rather UV detection. UV detection may be unable to separate out the different GBE constituents and as such, the GBE formulation is given a total TTL percentage, rather than a breakdown of constituents. With the increased accessibility to mass spectrometry, future products and studies may be able to quantify GBE such to provide the individual constituent concentrations.

One consideration for this study is that it used a cohort of 18–40 years old. GBE is proposed to protect against symptoms of age-related cognitive decline. This is likely to only appear in a population significantly older than that used in this study. The effect this may therefore have on clinical outcomes is unknown, with future studies planned for different population groups (including over 40 years of age). However, this being the first trial for this product, we decided to commence with a young healthy population so not to induce potential for confounding factors such as medications and/or adverse health conditions.

The apparent differences between GBE products may also contribute to the variability in the reported efficacy of GBE. Despite the proven beneficial effects of GBE, there are also studies that have shown no benefits of GBE [19, 20]. However, this is not uncommon when studying a natural supplement, with trial aspects including the supplement source, supplement dose, population used, and trial duration, all able to affect the outcome of a trial.

### Conclusion

In conclusion, the aim of this study was to assess the total absorption profile (AUC), C_max_ and T_max_ of 2 different GBE formulations in healthy participants over a 24-h period. This study showed that compared with a dose of standard GBE, an equivalent single dose of liposomal GBE resulted in a 1.9 and 2.2-fold increase in absorption (measured as AUC) and a 1.7 and 1.2-fold increase in C_max_ (Table 2, Fig. 1A  and B) for GB and GC respectively. Future directions for studies from here may include longer term dosing studies to work out if there is an accumulation effect using liposomal formulations, dosing trials to work out the best method for optimising plasma and cellular levels of GBE and progressing into clinical trials to work out the efficacy of the trial product using the optimal dosing schedule.

## Data Availability

The datasets used and/or analysed during the current study are available from the corresponding author on reasonable request. This is due to this study being a commercially funded study and the data and all intellectual property owned by the sponsor.

## Abbreviations

GBE: *Ginkgo biloba* Extracts
TTL: Terpene trilactones
GA: Ginkgolide A
GB: Ginkgolide B
GC: Ginkgolide C
GJ: Ginkgolide J
BB: Bilobalide
T_max_: Maximum concentration
LC–MS/MS: Liquid-chromatography tandem mass spectrometry
AUC: Area under the curve
BLD: Below limit of detection ()
